# Modified triglyceride-glucose index indices are reliable markers for predicting risk of metabolic dysfunction-associated fatty liver disease: a cross-sectional study

**DOI:** 10.3389/fendo.2023.1308265

**Published:** 2024-01-22

**Authors:** Ae Hee Kim, Da-Hye Son, Yong-Jae Lee

**Affiliations:** Department of Family Medicine, Gangnam Severance Hospital, Yonsei University College of Medicine, Seoul, Republic of Korea

**Keywords:** TyG index, TyG-WC, TyG-BMI, MAFLD, metabolic dysfunction

## Abstract

**Introduction:**

Metabolic dysfunction–associated fatty liver disease (MAFLD) is newly proposed nomenclature, and its diagnosis involves an algorithm that can be complicated and impractical for clinicians in real-world clinical settings. Thus, we investigated the association between MAFLD and modified triglyceride-glucose index (TyG) indices to find a more concise, feasible method for predicting MAFLD in everyday clinical care.

**Methods:**

Data were obtained from people who voluntarily underwent health check-ups at the Health Promotion Centre of Gangnam Severance Hospital, Yonsei University College of Medicine, from January 2017 to October 2020. Four indices were analyzed: TyG-body to mass index (BMI), TyG-waist circumference (WC), TyG, and the fatty liver index (FLI). The odds ratios for MAFLD according to each index were calculated using multiple logistic regression analyses, and the receiver operating characteristics curve (ROC) and area under the ROC were obtained to find the predictive powers of each index.

**Results:**

The final number of study participants was 22,391, 8,246 with MAFLD and 14,145 without MAFLD. The odds ratios (95% confidence intervals) from TyG-WC and TyG-BMI after adjusting for confounding variables were 12.484 (9.962–15.644) and 12.494 (9.790–15.946), respectively, for quartile 2, 54.332 (43.131–68.442) and 51.580 (40.495–65.699) for quartile 3, and 165.804 (130.243–211.076) and 128.592 (100.601–164.371) for quartile 4. The area under the ROC curve values for TyG-WC and TyG-BMI were 0.862 (0.857–0.867) and 0.867 (0.862–0.872), respectively.

**Conclusion:**

The modified TyG indices are highly reliable markers for predicting MAFLD that clinicians can easily and practically apply in everyday, real-world, clinical care settings.

## Introduction

Recently, international experts have proposed a change of nomenclature concerning fatty liver disease (1). The disease that has been called non-alcoholic fatty liver disease (NAFLD) for decades (2), is now proposed to be renamed metabolic dysfunction–associated fatty liver disease (MAFLD) (1, 3). Aside from simply being a hepatic disease, NAFLD is also known to be associated with a variety of extra-hepatic co-morbidities such as chronic kidney disease, osteoporosis, polycystic ovarian syndrome, and extra-hepatic cancers such as bladder cancer (4, 5). Due to these associations, MAFLD as a novel nomenclature may be more appropriate than NAFLD. The key feature defining NAFLD is a negative criterion that excludes alcohol-related liver disease, whereas MAFLD is defined as a set of positive criteria, including alcohol-related liver problems, that emphasize metabolic dysfunction and abnormalities. Thus, a patient can be identified with both MAFLD and NAFLD or might not be identified at all if the criteria for only one or the other are applied, such as in cases with an alcohol-related origin accompanied by metabolic abnormalities. Although controversy about the paradigm shift to MAFLD is ongoing, much research is being published about the clinical importance of MAFLD (6–8).

Kim et al. published a study that evaluated the prevalence of fatty liver disease (FLD) when the definitions for NAFLD and MAFLD were applied, along with the association between each condition and cardiovascular disease (CVD) risk (9). Based on a nationwide health screening database with a median follow-up of 10.1 years among Koreans, and compared with the no-FLD group as the reference, the risk of CVD events was significantly higher in the MAFLD-only and both-FLD groups than in the NAFLD-only group. Another group from the United States came to a similar conclusion about the superiority of MAFLD, compared with NAFLD, in predicting CVD risk (10).

However, diagnosis of MAFLD requires adherence to an algorithm that might be too complex or impractical for clinicians in real-world settings. The diagnostic algorithm for identifying MAFLD involves the presence of histological, imaging, or blood biomarker evidence of fat accumulation in the liver (hepatic steatosis) along with one of the following: overweight/obesity, type 2 diabetes, or evidence of metabolic dysregulation. Metabolic dysregulation is defined as the presence of at least two metabolic risk abnormalities, such as abdominal obesity, elevated blood pressure, increased triglyceride levels, reduced high density lipoprotein (HDL) cholesterol levels, prediabetes, insulin resistance, or an elevated high-sensitivity C-reactive protein level (3). Consequently, a concise and straightforward method for predicting the risk of MAFLD is needed to enable more practical early detection in everyday clinical care.

Although the precise pathophysiology for MAFLD is unclear, one mechanism of metabolic dysfunction is known to derive from insulin resistance (11). The triglyceride-glucose (TyG) index has been emerging as a simple and inexpensive tool for measuring insulin resistance and accurately predicting the risk of metabolic syndrome and NAFLD (12, 13), and some recent studies have found similar results with MAFLD (14, 15). Furthermore, a growing body of research is focusing on modified TyG indices that incorporate waist circumference (WC) or body mass index (BMI) to improve the predictive performance for metabolic diseases (16, 17). To the best of our knowledge, very few published studies have explored the relationship between modified TyG indices and MAFLD (18). Thus, we aimed to find more feasible ways to predict MAFLD in clinical settings by analyzing the reliability of modified TyG indices in predicting the risk of MAFLD.

## Methods

### Study population

**Figure 1:**
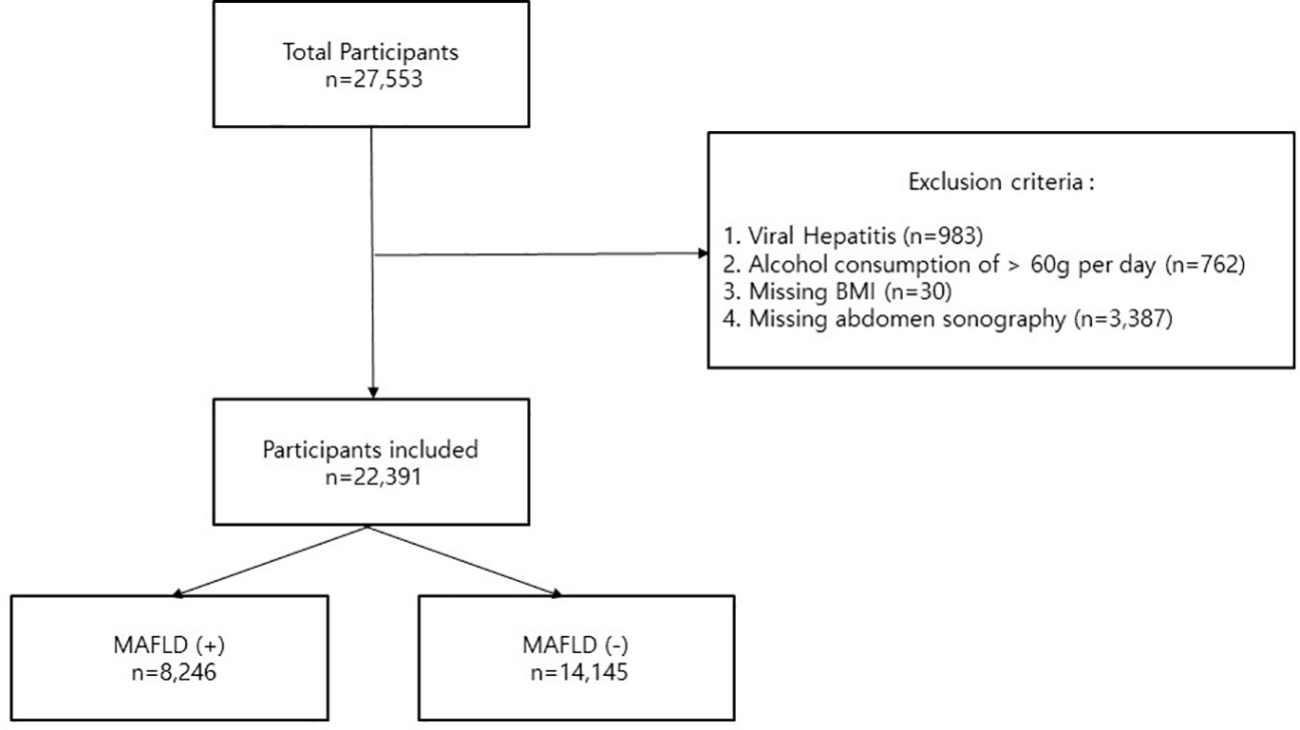
Flow chart of exclusion criteria and all study participants.

This cross-sectional study used a database obtained from the Health Promotion Centre of Gangnam Severance Hospital, Yonsei University College of Medicine. This database covers people who came in for regular health check-up examinations between January 2017 and October 2020. From a total of 27,553 participants, we excluded those with viral hepatitis (positive results for the hepatitis B virus surface antigen and/or anti-hepatitis C virus antibodies) (n=983), with alcohol consumption > 60g per day (n=762), missing BMI data (n=30), or missing abdomen ultrasound results (n=3,387). After excluding those participants, the final number of participants included in this study was 22,391 (**Figure 1**). Participation in the study was voluntary, and written informed consent was obtained from all participants. The Declaration of Helsinki was followed, and this study was approved by the Institutional Review Board of Yonsei University College of Medicine, Seoul, Korea (IRB number: 3-2021-0093).

### Data collection

The medical and social histories of each participant were acquired using self-response questionnaires that included questions about physical activity, smoking, and alcohol consumption. Regular exercise was classified as regular if exercise was conducted at least three times per week. Smoking status was categorized as non-smoker, former smoker, and current smoker.

Anthropometric values were measured by trained medical staff following standardized procedures. Body weight was measured to the nearest 0.1kg in light outdoor clothing, and height was measured to the nearest 0.1cm without shoes. WC was measured to the nearest 0.1cm midway between the lower rib margin and the iliac crest in a horizontal plane following a normal expiration. BMI was calculated by dividing weight by the square of height in meters (kg/m^2^). Systolic blood pressure (SBP) and diastolic blood pressure (DBP) were measured on the right arm of each participant after a 5-minute rest and using a standard mercury sphygmomanometer (Baumanometer, W.A. Baum Co., Inc., Copiague, NY, USA).

To measure the biochemical parameters, blood samples were drawn from the antecubital veins of participants after a 12-hour overnight fast. Fasting plasma glucose, glycosylated hemoglobin (HbA1c), total cholesterol, triglyceride (TG), high-density lipoprotein cholesterol (HDL-C), low-density lipoprotein cholesterol (LDL-C), aspartate aminotransferase (AST), alanine aminotransferase (ALT), alkaline phosphatase (ALP), and gamma-glutamyl transferase (γ-GT) values were all obtained through a chemistry analyzer using enzymatic methods (Hitachi 7600, Hitachi Co., Tokyo, Japan). Homeostasis model assessment-insulin resistance (HOMA-IR) scores were calculated as [(fasting insulin (μIU/mL)) x (fasting glucose (mg/dL))/405] (19).

Among the comorbidities, hypertension (HTN) was defined as SBP ≥ 140 mmHg, DBP ≥ 90 mmHg, or current use of anti-hypertensive medication. Fasting plasma glucose of ≥ 126mg/dL or current use of anti-diabetic drugs or insulin defined type 2 diabetes mellitus (T2DM). Dyslipidemia (DL) was defined as TG ≥ 150 mg/dL, HDL-C < 50 mg/dL, or current use of lipid-lowering medication.

### Indices: TyG index, modified TyG-related parameters, and FLI

The TyG index was calculated using the following formula: ln [fasting serum TG (mg/dL) × fasting plasma glucose (mg/dL)/2] (20). TyG-BMI and TyG-WC were calculated as [TyG index × BMI] and [TyG × WC], respectively. The fatty liver index (FLI) value was obtained through the following formula: (e^0.953*loge(triglycerides)+0.139*BMI + 0.718*loge(γ–GT) + 0.053*WC – 15.745^)/(1 + e^0.953*loge (triglycerides)+0.139*BMI + 0.718*loge(γ–GT) + 0.053*WC – 15.745^) * 100 (21).

### Definition of MAFLD

Hepatic steatosis was detected by an abdominal ultrasonography scan conducted by two radiologists uninformed of the aims of this study. Scans were performed with a 3.5-MHz transducer (HDI 5000, Philips, Bothell, WA, USA), and the reproducibility of inter- and intra-operator variation coefficients were 6.8% and 4.3%, respectively.

MAFLD was defined as the presence of hepatic steatosis plus one of the following criteria (3): overweight/obesity defined as BMI ≥ 23 kg/m^2^, T2DM, or evidence of metabolic dysregulation. Metabolic dysregulation was defined as the presence of two or more metabolic risk abnormalities: WC ≥ 90 cm in males and ≥ 80 cm in females, blood pressure ≥ 130/85 mmHg or specific drug treatment, TG ≥ 150 mg/dl or specific drug treatment, HDL-C < 40 mg/dl for males and < 50 mg/dl for females or specific drug treatment, fasting serum glucose ≥ 126 mg/dl, and HOMA-IR score ≥ 2.5.

### Statistical analysis

In comparing the clinical characteristics of the study population, we used the independent two-sample student’s t-test for continuous variables and the chi-square test for categorical variables. The data are expressed as the mean ± standard deviation. The odds ratios (ORs) and 95% confidence intervals (CIs) for MAFLD according to each index were calculated with multiple logistic regression analyses after adjusting for potentially confounding variables. The confounding variables were age, sex, AST, ALT, γ-GT, SBP, DBP, HTN, T2DM, DL, smoking, and regular exercise. The indices tested for their association with MAFLD were TyG, FLI, TyG-WC, and TyG-BMI. To test the prediction of MAFLD by index, we obtained the receiver operating characteristics (ROC) curves and conducted area under the ROC (AUROC) analyses. The statistical analyses were calculated by Statistical Package for Social Sciences software (SPSS version 26; IBM Corp., Armonk, NY, USA), and p-values of < 0.05 were considered to be statistically significant.

## Results

**Table 1** summarizes the characteristics of the study population according to whether they had MAFLD (n=8,246), or not (n=14,145). Compared with those in no MAFLD group, those with MALFD were older (51.50 ± 11.5 years vs. 47.9 ± 12.9 years), and predominantly male (n=5,737, 69.6%). BMI, WC, SBP, DBP, TyG, FLI, TyG-WC, and TyG-BMI were all significantly higher in the MAFLD group. Biochemistry measurements showed similar patterns: the MAFLD group had significantly higher levels of fasting plasma glucose, HbA1c, total cholesterol, TG, LDL-C, AST, ALT, γ-GT, and HOMA-IR and lower levels of HDL-C than the no MAFLD group. The MAFLD group had higher proportions of comorbidities (HTN, T2DM, and DL) and smoked more and exercised less than the no MAFLD group.

**Table 1:** Baseline characteristics of the study population.

Total n=22,391	Without MAFLD n = 14,145	MAFLD n = 8246	*p*-value^*^
Demographics			
Age (years)	47.9 ± 12.9	51.50 ± 11.5	<0.001
Male (%)	5610 (39.7)	5737 (69.6)	<0.001
Female (%)	8535 (60.3)	2509 (30.4)	<0.001
Anthropometrics			
Body mass index (kg/m^2^)	22.5 ± 2.9	26.5 ± 3.3	<0.001
Waist circumference (cm)	77.1 ± 9.2	89.3 ± 8.9	<0.001
Systolic blood pressure (mmHg)	118.7 ± 12.6	123.9 ± 12.1	<0.001
Diastolic blood pressure (mmHg)	70.1 ± 8.7	72.8 ± 9.1	<0.001
Biochemistry			
Fasting plasma glucose (mg/dL)	95.5 ± 14.8	107.6 ± 25.0	<0.001
Hemoglobin A1c (%)	5.5 ± 0.5	5.9 ± 0.9	<0.001
Total cholesterol (mg/dL)	202.7 ± 36.9	207.3 ± 42.1	<0.001
Triglyceride (mg/dL)	90.0 (68.0, 123.0)	145.0 (105.0, 202.0)	<0.001
HDL-C (mg/dL)	61.0 ± 13.6	51.0 ± 11.0	<0.001
LDL-C (mg/dL)	125.2 ± 29.6	133.7 ± 33.0	<0.001
AST (IU/L)	27.4 ± 16.0	32.3 ± 19.3	<0.001
ALT (IU/L)	22.3 ± 15.9	36.2 ± 25.8	<0.001
Alkaline phosphatase (IU/L)	70.0 ± 25.3	77.0 ± 21.0	<0.001
γ-GT (IU/L)	17.0 (12.0, 25.0)	30.0 (21.0, 47.0)	<0.001
HOMA-IR (*μ*U/L)	1.60 ± 1.22	2.97 ± 2.12	<0.001
Comorbidities			
HTN medication (%)	1622 (43.1)	2144 (56.9)	<0.001
DM medication (%)	430 (33.1)	868 (66.9)	<0.001
DL medication (%)	1414 (47.2)	1579 (52.8)	<0.001
Lifestyle			
Current smoker (%)	1763 (49.7)	1787 (50.3)	<0.001
Regular exercise (%)	10039 (64.2)	5597 (35.8)	<0.001
Parameters			
TyG	8.40 ± 0.49	8.97 ± 0.55	<0.001
FLI	9.79 (4.28, 22.92)	45.76 (27.61, 67.32)	<0.001
TyG-WC	649.2 ± 100.3	802.7 ± 102.1	<0.001
TyG-BMI	189.3 ± 30.4	237.5 ± 34.7	<0.001

Data are expressed as the mean ± SD or percentage. ^*^p-values were calculated using student t-test or the chi-squared test. HDL-C, high-density lipoprotein cholesterol; LDL-C, low-density lipoprotein cholesterol; AST, aspartate transferase; ALT, alanine transferase; γ-GT γ-glutamyl transferase; HOMA-IR, homeostatic model assessment for insulin resistance; HTN, hypertension; DM, diabetes mellitus; DL, dyslipidemia.

**Table 2** presents the ORs and 95% CIs of the different indices in association with MAFLD, categorized into quartiles. The ORs for the lowest quartiles (Q1) of all indices in all models were set as 1.00 for purpose of comparison. In model 1, which was unadjusted, the TyG index showed increasing ORs as the quartiles increased; the OR for Q2 was 3.188 (2.861–3.553), Q3 7.908 (7.127–8.775), and Q4 23.386 (21.014–26.026). In model 2, which was adjusted for age and sex, the ORs of the TyG index showed the same pattern: Q2 2.690 (2.409–3.003) and Q4 16.311 (14.607–18.213). In model 3, which was adjusted for many possibly confounding variables, the ORs still displayed the same pattern: Q2 2.403 (2.140–2.699), and Q4 10.977 (9.747–12.364). The ORs of the FLI, TyG-WC, and TyG-BMI also increased with their corresponding quartiles in all three models. In particular, the ORs of TyG-BMI were 12.494 (9.790–15.946) in Q2, 51.580 (40.495–65.699) in Q3, and 128.592 (100.601–164.371) in Q4 after adjusting for all the confounding variables.

**Table 2:** Odds ratios for MAFLD according to the quartiles of each parameter.

		Model 1			Model 2			Model 3		
		OR	95% CI	*p*-value	OR	95% CI	*p*-value	OR	95% CI	*p*-value
**TyG**	Q1	1	–	<.0001	1	–	<.0001	1	–	<.0001
	Q2	3.188	2.861-3.553	<.0001	2.690	2.409-3.003	<.0001	2.403	2.140-2.699	<.0001
	Q3	7.908	7.127-8.775	<.0001	6.040	5.428-6.721	<.0001	4.699	4.196-5.263	<.0001
	Q4	23.386	21.014-26.026	<.0001	16.311	14.607-18.213	<.0001	10.977	9.747-12.364	<.0001
**FLI**	Q1	1	–	<.0001	1	10.922-17.241	<.0001	1	–	<.0001
	Q2	12.676	10.120-15.877	<.0001	13.722	51.979-82.274	<.0001	12.203	9.701-15.351	<.0001
	Q3	55.957	44.882-69.766	<.0001	65.395	176.470-282.411	<.0001	53.428	42.368-67.374	<.0001
	Q4	181.927	145.504-227.468	<.0001	223.242	10.922-17.241	<.0001	164.761	129.173-210.154	<.0001
**TyG-WC**	Q1	1	–	<.0001	1	–	<.0001	1	–	<.0001
	Q2	11.491	9.292-14.211	<.0001	13.964	11.257-17.323	<.0001	12.484	9.962-15.644	<.0001
	Q3	47.059	38.230-57.928	<.0001	69.249	55.586-86.270	<.0001	54.332	43.131-68.442	<.0001
	Q4	161.102	130.472-198.923	<.0001	257.928	205.427-323.847	<.0001	165.804	130.243-211.076	<.0001
**TyG-BMI**	Q1	1	–	<.0001	1	–	<.0001	1	–	<.0001
	Q2	15.080	11.953-19.026	<.0001	13.240	10.476-16.733	<.0001	12.494	9.790-15.946	<.0001
	Q3	74.596	59.376-93.716	<.0001	61.066	48.430-76.999	<.0001	51.580	40.495-65.699	<.0001
	Q4	227.445	180.618-286.411	<.0001	185.041	146.411-233.863	<.0001	128.592	100.601-164.371	<.0001

Model 1: unadjusted.

Model 2: adjusted for age and gender.

Model 3: adjusted for age, gender, AST, ALT, _γ_GT, SBP, DBP, HTN, DM, DL, smoking, and exercise.

**Figure 2:**
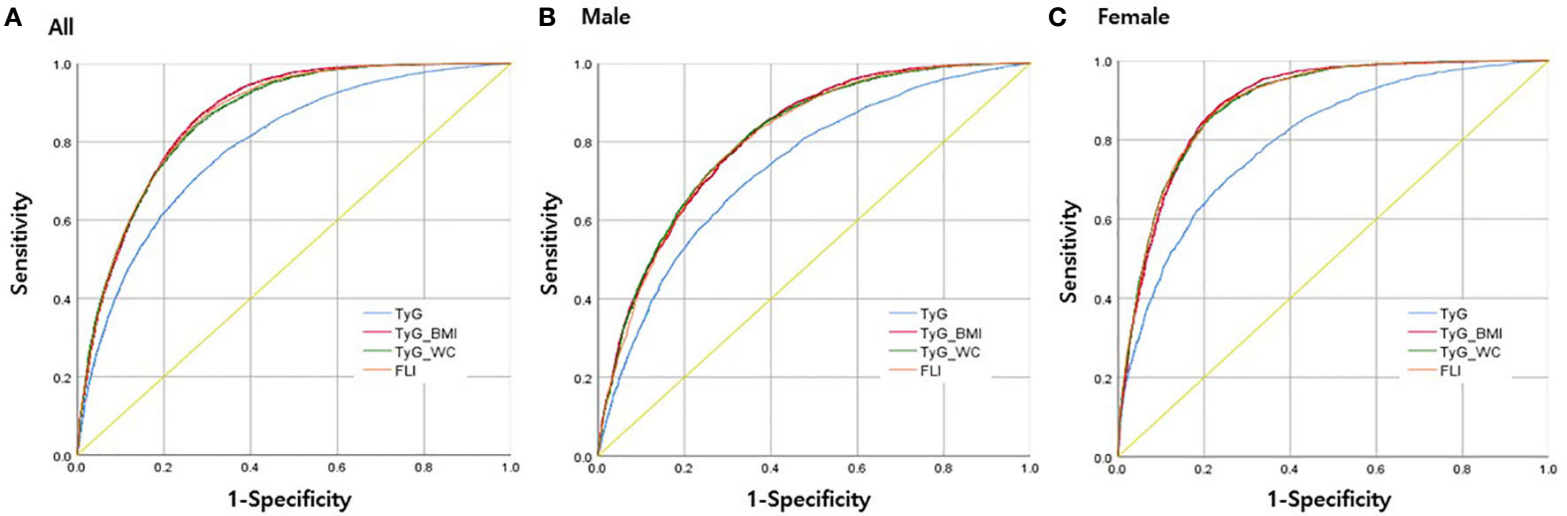
Receiver operating characteristics (ROC) curves for each parameter in predicting MAFLD **(A)** in all participants, **(B)** in men only, and **(C)** in women only.

**Figure 2** illustrates the ROC curves of each index in predicting MAFLD in all participants, males, and females. The AUROC values for TyG, FLI, TyG-WC, and TyG-BMI were 0.790 (0.783–0.796), 0.864 (0.859–0.869), 0.862 (0.857–0.867), and 0.867 (0.862–0.872), respectively (**Table 3**). In males, the AUROC values for the indices in the same order were 0.736 (0.727–0.746), 0.807 (0.799–0.816), 0.810 (0.802–0.818), and 0.812 (0.803–0.820). In females, the values were 0.801 (0.790–0.811), 0.895 (0.888–0.902), 0.894 (0.887–0.901), and 0.895 (0.888–0.901). The predictive power of modified TyG indices was thus especially superior in females.

**Table 3:** Area under the receiver operating characteristic curve (AUROC) values of each parameter in prediction of MAFLD, according to all participants, males only, or females only.

Parameters	AUROC	95% CI	Sensitivity (%)	Specificity (%)	PPV (%)	NPV (%)
All participants						
*TyG*	0.790	0.783-0.796	75.2	68.4	58.1	82.5
*FLI*	0.864	0.859-0.869	87.8	69.6	62.7	90.7
*TyG-WC*	0.862	0.857-0.867	83.9	72.8	64.2	88.5
*TyG-BMI*	0.867	0.862-0.872	86.3	72.3	64.4	90.0
Male only						
*TyG*	0.736	0.727-0.746	68.2	67.3	68.1	67.3
*FLI*	0.807	0.799-0.816	78.1	68.8	71.9	75.4
*TyG-WC*	0.810	0.802-0.818	81.6	65.5	70.7	77.6
*TyG-BMI*	0.812	0.803-0.820	81.9	65.1	70.6	77.8
Female only						
*TyG*	0.801	0.790-0.811	98.6	46.3	35.0	99.1
*FLI*	0.895	0.888-0.902	86.8	77.8	53.4	95.2
*TyG-WC*	0.894	0.887-0.901	86.5	77.7	53.2	95.1
*TyG-BMI*	0.895	0.888-0.901	88.9	76.4	52.5	95.9

## Discussion

Our results demonstrate that the TyG index is an extremely reliable predictive marker for MAFLD, and the modified TyG indices have even more superior in their predictive power, especially in females.

Each modified TyG index is easily accessible because the parameters, WC, and BMI, can be easily measured. Additionally, plasma TG and glucose are biochemical values routinely measured in primary care clinics. The more traditional methods for predicting FLD have some limitations. When measuring insulin resistance, the gold standard is the hyperinsulinemic-euglycemic clamp method, but it is rarely used in clinical settings due to high cost and low accessibility (22). The HOMA-IR is another method for measuring insulin resistance, but it is being suggested to be inferior to the TyG index (17). For diagnosing hepatic steatosis, ultrasonography is expensive, time-consuming, and requires professional medical personnel, and liver histology is extremely invasive. The FLI is composed of readily available parameters, and our study has shown that its predictive power is also strong. However, the formula for acquiring FLI values is complex, which discourages clinicians from using it in clinical settings. Therefore, our results could revolutionize how primary care physicians screen for MAFLD risk prior to the development of complications such as CVD, DM, and other comorbidities.

Previous studies have shown results similar to ours. In one cross-sectional study of 1,727 adults, the AUROC values for predicting MAFLD were 0.822 for TyG-BMI and 0.832 for TyG-WC (18). In another study, a cohort of 2,056 participants with an average follow-up of 2.5 ± 0.5 years showed that the TyG index positively correlated with the risk of incident MAFLD with a hazard ratio 1.784 (95% CI 1.383–2.302) (14). Our results are consistent with those previous studies in terms of the positive association between the TyG index and MAFLD. However, to the best of our knowledge, only one previous study used the modified TyG indices to predict MAFLD, and that study had a relatively small sample of 1,727 participants, whereas our study used a large sample (n=22,391) and thus has stronger statistical reliability.

Although the precise underlying mechanisms connecting the modified TyG indices with MAFLD are not clearly known, some plausible explanations support our results. The TyG index represents insulin resistance as derived from both the liver and muscle. Plasma TG interferes with glucose metabolism in muscle and eventually leads to insulin resistance. In addition, increased plasma TG from visceral fat leads to an increase of free fatty acids in the liver, which reduces insulin sensitivity of hepatic origin (23–25). Furthermore, the modified parameters of the TyG-BMI and TyG-WC are even stronger predictors of MAFLD than the TyG index alone, probably because they include body composition in the formula. BMI is an established marker for general obesity, and WC represents visceral fat deposition, which is associated with insulin resistance, metabolic dysfunction, and hepatic steatosis (26–28). Because MAFLD is diagnosed using positive criteria that include components of overweight and obesity, it is obvious that modifying the TyG index with WC or BMI would increase its ability to predict MAFLD. Furthermore, serum glucose, part of the TyG index, is highly associated with insulin resistance and DM, which might explain the high association between the modified TyG indices and MAFLD, which includes metabolic abnormalities in its definition (17).

In females, the predictive powers of the modified TyG indices were even greater than in the overall population, and other studies have also reported similar findings in the recent years. Li et al. investigated the association between the TyG index and NAFLD and demonstrated that the OR for NAFLD associated with the TyG index was significantly higher in females than males (females: OR 2.69, 95% CI 1.67–4.23; males: OR 2.09, 95% CI 1.59–2.76) (29). In another study that investigated a possible dose-response association between the TyG index and the risk of NAFLD, the TyG index had a stronger association with NAFLD in females than in males (females: OR 4.80, 95% CI 3.90–5.90; males: OR 2.97, 95% CI 2.55–3.46) (30). A plausible explanation for this discrepancy could stem from a complex of sex differences in adiposity and other metabolic risk factors in association with fatty liver diseases, including glucose and lipid metabolism, and insulin resistance (31).

Some issues from our study remain unresolved. First, although our study included a substantial number of participants, our population consisted solely of individuals of Korean descent. Consequently, our findings might not be applicable to other ethnic groups. Second, due to the study’s cross-sectional design, it was not possible to determine the cumulative incidence rate of MAFLD or establish a longitudinal connection between the modified TyG indices and the development of MAFLD. To validate our findings, additional prospective or longitudinal studies are necessary. Third, CRP values were not available in our dataset. Further studies with sufficient CRP data are required to validate our findings.

## Conclusion

In conclusion, the modified TyG indices were independently and positively associated with the risk of MAFLD and can identify subjects at risk using rapid, inexpensive, non-invasive, and practical methods in real-world clinical settings.

## Data availability statement

The raw data supporting the conclusions of this article will be made available by the authors, without undue reservation.

## Author contributions

AK: Conceptualization, Data curation, Writing – original draft, Writing – review & editing. D-HS: Conceptualization, Data curation, Supervision, Writing – original draft, Writing – review & editing. Y-JL: Conceptualization, Funding acquisition, Project administration, Writing – original draft, Writing – review & editing.
